# Risk factors for cancer-associated thrombosis in patients undergoing treatment with immune checkpoint inhibitors

**DOI:** 10.1007/s10637-019-00881-6

**Published:** 2019-12-10

**Authors:** Yosuke Ando, Takahiro Hayashi, Reiko Sugimoto, Seira Nishibe, Kaori Ito, Kenji Kawada, Yoshiaki Ikeda, Shigeki Yamada, Kazuyoshi Imaizumi

**Affiliations:** 1 Department of Clinical Pharmacy, Fujita Health University School of Medicine, 1-98 Dengakugakubo, Kutsukake-cho, Toyoake, 470-1192 Japan; 2 College of Pharmacy, Kinjo Gakuin University, Nagoya, 463-8521 Japan; 3 Department of Hematology, Fujita Health University School of Medicine, Toyoake, 470-1192 Japan; 4 Department of Medical Oncology, Fujita Health University School of Medicine, Toyoake, 470-1192 Japan; 5 Department of Respiratory Medicine, Fujita Health University School of Medicine, Toyoake, 470-1192 Japan

**Keywords:** Cancer-associated thrombosis, Venous thromboembolism, Arterial thromboembolism, Immune checkpoint inhibitor, Nivolumab, Pembrolizumab

## Abstract

*Purpose* Anticancer agents are known to increase cancer-associated thrombosis (CAT) onset. CAT onset rate is reported to be 1.92% in cisplatin-based therapy, 6.1% in paclitaxel plus ramucirumab combination therapy, and 11.9% in bevacizumab monotherapy. Because immune checkpoint inhibitors (ICIs) cause a sudden increase in T cell number, an association between administration of these drugs and increase in CAT incidence is likely. However, the extent to which ICI administration affects CAT incidence remains unclear. Further, risk factors for CAT incidence have not yet been identified. The present study investigated CAT incidence and associated risk factors in patients receiving ICI. *Methods* Patients administered nivolumab or pembrolizumab at Fujita Health University Hospital from April 2017 to March 2018 were enrolled. We collected retrospective data regarding age, sex, cancer type, BMI, medical history, laboratory data at treatment initiation, medications, and computed tomography (CT) interpretations from electronic medical records. *Results* We identified 122 eligible participants from 135 patients receiving nivolumab or pembrolizumab. Ten patients (8.2%) developed CAT. A history of venous thromboembolism (VTE) or arterial thromboembolism (ATE) was a risk factor for CAT incidence (odds ratio: 6.36, *P* = 0.039). A history of heart disease may be a risk factor for CAT incidence (odds ratio 6.56, *P* = 0.052). Significantly higher usage of antiplatelet and anticoagulant therapy was noted in patients who developed CAT (60%) than in those who did not (13.4%, *p* < 0.01). *Conclusion* High (8.2%) CAT incidence during ICI administration suggested that ICI is not associated with a lower blood clot risk than other anticancer agents investigated in previous studies. For patients with VTE, ATE, or heart disease history, it is crucial to consider the possibility of CAT even with antiplatelet therapy.

## Introduction

Improved quality and safety in cancer treatment, including the development of targeted anticancer agents, is improving survival rates in cancer patients. In many cases, patients die due to treatment complications instead of the cancer per se [1]. According to Khorana AA [2], cancer per se was the most common cause of death in cancer patients, followed by thrombosis. There are two types of thrombosis, venous thromboembolism (VTE) which includes pulmonary thromboembolism and deep vein thrombosis, and arterial thromboembolism (ATE) which includes cerebral infarction, myocardial infarction, and peripheral embolism. When thrombosis develops in cancer patients, it is referred to as cancer-associated thrombosis (CAT). Compared with patients without cancer, the onset rate of VTE or ATE in cancer patients is 4–7 times higher for VTE [3] and two times higher for ATE [4]. In addition, the onset rate of myocardial infarction has been reported to be three times higher in cancer patients than in patients without cancer [4].

Various risk factors such as age, sex, performance status (PS), and cancer type are reportedly associated with CAT onset. The type of anticancer agent may be an additional risk factor [5]. Previous studies have investigated only VTE onset rate, which is reportedly 1.92% with the use of regimens containing cisplatin (CDDP) [6], 3.8% with ramucirumab (Ram) alone, 6.1% with Ram + paclitaxel (PTX) combination therapy [7–11] and 11.9% with bevacizumab (Bev) alone [12]. In addition, a study that compared VTE onset rate in a CDDP-based treatment group (CDDP group) and a non-CDDP group showed that it was 1.67 times higher in the CDDP group [6]. On comparing standard antineoplastic therapy used with or without Bev, VTE onset rate was reported to be 1.33 times higher in the Bev group than in the non-Bev group [12]. In the case of nivolumab administration, only VTE incidence [13–20] has been reported and no reports on risk factors for CAT onset including ATE are present. Moreover, no reports on VTE or ATE onset rate during pembrolizumab administration are available.

It has been reported that the mechanism of CAT development in tumor-bearing patients involves microvesicles rich in tissue factors being released from neoplastic cells, promoting fibrin formation and platelet aggregation. Meanwhile, circulating mucins and P-selectins act to form platelet-rich microthrombi [21]. In a case report, Kunimasa et al. [22] suggested that the sudden increase of reactivated T cells immediately after pembrolizumab administration could be associated with CAT onset. Conventionally, although immune checkpoint inhibitors (ICIs) are considered less cardiotoxic, Johnson DB et al. [23] reported the cases of two patients with melanoma in whom fatal myocarditis developed following treatment with ipilimumab and nivolumab. A meta-analysis conducted by Wang DY et al. investigating the fatal toxic effects associated with ICI [24] showed that mortality in patients who developed cardiac or neurological disorders was 43%, which was compared with other side effects. Although the incidence of CAT leads to cardiovascular events, cardiac function disturbance can trigger blood clot development. In other words, the mechanism of CAT development during ICI administration to cancer patients may differ from that of CAT development in tumor-bearing patients proposed by Varki [21]. Therefore, understanding the risk of CAT incidence induced by ICI administration including risk factors for CAT onset is meaningful in assessing points of caution during clinical ICI administration.

## Materials and methods

### Subjects

Patients who underwent treatment at Fujita Health University Hospital from 1st April, 2017, to 31st March, 2018 with nivolumab or pembrolizumab for lung cancer, kidney cancer, stomach cancer, urothelial carcinoma, or malignant melanoma were enrolled in the study. Patients who developed thrombosis at the start of ICI administration were excluded.

### Investigations

This was a retrospective study to identify patients and associated variables from electronic medical records of Fujita Health University Hospital. CAT-positive patients who developed new blood clots within the period from the start of ICI administration to 3 months after it ended were classified as a P group, whereas CAT-negative patients were classified as an N group. Data on the following variables that may be the risk factor for CAT onset were extracted: pre-treatment age, sex, cancer type [5], blood type [25], BMI, thromboembolic disease history, heart disease history, diabetes history, blood transfusion history, surgery history, and radiation history. For blood biomarkers, pre-treatment white blood cell (WBC), hemoglobin (Hb), platelet (Plt) and D-dimer [26] values were collected. The use of antiplatelet therapy, anticoagulants, steroids, and erythropoietin drugs during ICI administration were included in the analysis. The pre-treatment Khorana VTE risk assessment score in the P group patients was calculated.

### Assessment

Blood clots were confirmed from the interpretation of CT and ultrasound imaging as well as medical charts. Khorana VTE risk assessment score was calculated based on cancer type, pre-treatment Plt count, Hb level, erythropoietin drug use, WBC count, and BMI.

### Statistical analysis

Non-normally distributed variables were described by the median and interquartile range. Non-parametric pairwise comparisons were performed using the Mann–Whitney U test. Univariate analysis was performed as an exploratory analysis of the risk factors. Subsequently, factors with hazard rates of <20% were incorporated in the multivariate logistic regression model. Hosmer–Lemeshow statistical test was used to verify the goodness of fit. The statistical analysis software used was SPSS Ver.22.0 (IBM Corporation, Armonk, NY, USA), and the significance level was set at less than 5%.

### Ethics

The study is in compliance with the Ethical Guidelines for Clinical Research and was approved by the Medical Research Institutional Review Board of Fujita Health University.

## Results

### Patients

There were 122 patients (85 in the nivolumab group and 37 in the pembrolizumab group); 10 patients were included in the P group (8.2%) and 112 patients were included in the N group (91.8%). The P group comprised 6 patients in the nivolumab group and 4 patients in the pembrolizumab group. Incidentally, the N group comprised 79 patients in the nivolumab group and 33 patients in the pembrolizumab group.

CAT incidence in the nivolumab group and the pembrolizumab group was 7.1% and 10.8%, respectively, with no significant differences between the two groups (*p* = 0.74). In addition, the pre-treatment patient background in the P group and N group was compared. Statistically significant differences were found between thromboembolic disease history, heart disease history, and pre-treatment Plt (Table 1). Medications taken during ICI administration were investigated, and it was found that use of antiplatelet drugs was higher in the P group than in the N group (Table 2).

**Table 1:** Patient background (before chemotherapy)

	P group (*n* = 10)	N group (*n* = 112)	*P* value
Age (years)	75.0 (62.0–78.0)	69.5 (61.0–74.0)	0.37
Sex (M/W)	10/0	81/31	0.12
BMI (kg/ m^2^)	21.2 (19.0–23.3)	21.3 (19.4–23.8)	0.75
Blood type O (%)	20.0	30.4	0.74
Stomach cancer (%)	20.0	12.5	0.85
Thromboembolic disease history (%)	70.0	11.6	<0.001
Heart disease history (%)	80.0	18.8	<0.001
Diabetes history (%)	40.0	15.2	0.12
Blood transfusion history (%)	80.0	55.4	0.24
Surgery history (%)	80.0	63.4	0.48
Radiation history (%)	50.0	48.2	0.82
Plt (×10^4^/μL)	17.0 (15.2–22.05)	24.0 (20.9–30.9)	0.014
WBC (×10^3^/μL)	6.2 (4.7–8.1)	6.7 (5.3–8.5)	0.50
Hb (g/dL)	11.9 (11.4–13.3)	11.7 (10.0–12.9)	0.23
D-dimer	2.3 (1.075–3.1)	1.25 (0.8–2.5)	0.17

**Table 2:** Patient background (during chemotherapy)

	*P* group (*n* = 10)	*N* group (*n* = 112)	*P* value
Medication			
Antiplatelet (%)	60.0	13.4	<0.001
Steroids (%)	20.0	23.2	0.87
Antithyroid (%)	10.0	16.1	0.96

### Risk factors

We performed univariate analysis on factors that had previously been reported as risk factors. Risk factors with a significance level < 20% were thromboembolic disease history, heart disease history, diabetes history, blood transfusion history, and antiplatelet drugs. Multivariate analysis was performed with these five risk factors. Only thromboembolic disease history was determined to be a significant risk factor (*P* = 0.039). We found that heart disease history could be a risk factor (*P* = 0.052) (Table 3).

**Table 3:** Risk factors for CAT incidence

	Univariate analysis		Multivariate analysis	
	Odds ratio (95% CI)	*P* value	Odds ratio (95% CI)	*P* value
Age	1.02	0.56		
	(0.96**–**1.086)			
BMI	0.98	0.82		
	(0.80**–**1.20)			
Stomach cancer	1.75	0.51		
	(0.34**–**9.09)			
Thromboembolic disease history	17.77	<0.001	6.36	0.039
	(4.082**–**77.35)		(1.10**–**36.71)	
Heart disease history	17.33	0.001	6.56	0.052
	(3.43**–**87.63)		(0.99**–**43.71)	
Diabetes history	3.73	0.060	1.102	0.915
	(0.95**–**14.61)		(0.184**–**6.61)	
Blood transfusion history	2.78	0.16	3.671	0.163
	(0.68–11.45)		(0.592–22.78)	
Surgery history	2.31	0.30		
	(0.47**–**11.40)			
Taking antiplatelet drugs	9.70	0.001	2.28	0.358
	(2.45–38.44)		(0.39–13.14)	

Predictive ability of the final model was quantified using the Hosmer–Lemeshow test for goodness of fit; *P* = 0.696

### Background of patients developing CAT

The background factors in the 10 patients who developed CAT are shown in Table 4. The median age was 75 years. Lung cancer was the most common type (six cases), followed by stomach cancer (three cases) and kidney cancer (one case). The median number of days to CAT onset was 90.0 days, with a minimum of six days and a maximum of 178 days. In terms of ICI, 6 patients received nivolumab and four received pembrolizumab. In terms of CAT, 5 patients developed ATE, four developed VTE, and one developed both ATE and VTE. Following ICI administration, approximately 4.9% (6/122 patients) developed ATE and approximately 4.1% (5/122 patients) developed VTE. D-dimer level in CAT-positive patients was investigated. In the 7 patients with D-dimer measurements, the biomarker level increased in the period from before ICI administration to CAT onset. The median increment level was 2.2, with a minimum of 0.7 and a maximum of 30.8. In the remaining 3 patients, D-dimer was not measured. After calculating Khorana score, we found that the VTE risk prediction score in all 10 patients was 2 or 1.

**Table 4:** List of CAT patients

No.	Age	Type of cancer	ICI	line	Number of days	Type of Cat	Part	D-dimer (before)	D-dimer (At the time)	Khorana score
1	43	Lung	Nivolumab	5th	22	VTE	Left brachiocephalic vein	5.5	6.2	2
2	71	Lung	Nivolumab	2nd	117	ATE	Myocardial infarction	N.D.	10.5	1
3	78	Kidney	Nivolumab	4th	178	ATE	Cerebral infarction	1.3	N.D.	1
4	79	Stomach	Nivolumab	3rd	70	VTE	Superior mesenteric vein	2.1	3.3	2
5	57	Barrett’s esophagus	Nivolumab	4th	54	ATE	Cerebral infarction	2.5	3.7	2
6	74	Stomach	Nivolumab	3rd	6	ATE	Cerebral infarction	2.8	7.3	2
7	59	Lung	Pembrolizumab	1st	111	VTE	Pulmonary embolism in the right femoral vein	0.4	17.4	1
8	78	Lung	Pembrolizumab	1st	37	ATE VTE	Cerebral infarction in the lower extremity veins Pulmonary embolism	4.0	34.8	2
9	76	Lung	Pembrolizumab	1st	150	VTE	Left femoral vein: external iliac vein	0.3>	2.5	1
10	79	Lung	Pembrolizumab	3rd	110	ATE	Cerebral infarction	N.D.	N.D.	1

## Discussion

The status of CAT incidence following ICI administration remains unclear, with insufficient current evidence. The present study showed that CAT incidence with nivolumab and pembrolizumab treatment was 7.1% and 10.8%, respectively, in clinical practice. These values were not lower than the CAT onset rates with Ram or Bev (VEGF inhibitors) treatment, which were 3.8% [7, 8] and 11.9% [13], respectively. Therefore, ICI administration and CAT onset may be related. In addition, to explore risk factors for CAT onset during ICI administration, we performed univariate analysis to determine the significantly different factors; significantly different variables in Table 1 include drugs closely related to blood clot formation and variables previously reported as risk factors. Candidate factors were identified and multivariate logistic regression analysis was performed. Our analysis showed that thromboembolic disease history was a risk factor for blood clot formation and heart disease history a potential risk factor. Khorana AA et al. investigated VTE risk in cancer patients [5] and showed that clinical risk factors such as cancer-related, treatment-related, and patient-related factors as well as candidate laboratory biomarkers were associated with for cancer-related VTE. They hypothesized that patient-related factors including ATE-related complications and VTE history were risk factors for VTE in cancer patients. Our findings in the present study are similar to the above previously reported data. Although many of the risk factors for VTE described by Khorana AA et al. [5] were not identified in the present study, we found that thromboembolic disease history and heart disease history were associated with CAT onset during ICI administration. This suggests the need for more frequent examinations related to blood clots when administering ICI to patients with thromboembolic disease history and heart disease history. In addition, Khorana AA et al. [5] reported that D-dimer was a risk factor for VTE in cancer patients. It has been reported that if the level increases by >1.44 μg/mL compared to that before treatment initiation, VTE onset is more likely [27]. Among our patients that developed CAT, D-dimer was measured in 7 patients. The levels increased in these patients in the period between ICI treatment initiation to CAT occurrence with a median increment level of 2.2, a minimum of 0.7, and a maximum of 30.8. Among these seven patients, only 4 showed an increase of >1.44 μg/mL in the D-dimer level. According to Stein PD et al. [28], D-dimer is inadequate to determine positive blood clot formation. Even if D-dimer was measured in routine medical examination, it would not be a predictive factor for blood clot formation.

ATE incidence in the present study following ICI administration was 4.9%. This was higher than the ATE incidence following Bev treatment (3.8%) [29]. Therefore, it appears crucial to focus on ATE onset when administering ICIs, as is the case for VEGF inhibitors. Because ATE has a high mortality risk when treatment is delayed, we recommend advanced collaboration with Departments of Strokology and Cardiology when administering ICIs to high-risk patients.

Further, we investigated the effects of platelets, which are related to blood clot formation, using the pre-treatment Plt count; levels in the P and N groups were compared; the Plt count in the N group was significantly higher than that in the P group. Khorana AA et al. studied VTE risk in cancer patients [5] and listed a pre-chemotherapy platelet count of ≥350,000/μL as one of the risk factors. Therefore, the difference in Plt count between the two groups in the present study may be less likely to be associated with the outcome. The lower Plt count in the P group compared with that in the N group could be attributed to the proportion of patients using antiplatelet drugs in the P group (60%), which was considerably higher than that in the N group (13.4%). Even if platelet aggregation had been controlled at treatment initiation, ICI administration itself could have led to CAT incidence in patients with thromboembolic disease history and heart disease history.

For CAT onset prediction, the Khorana score in the P group was calculated. We found that 5 out of 10 cases scored 2 points, whereas the other cases scored 1 point. Khorana score quantifies prediction of VTE risk before administration of anticancer agents in tumor-bearing patients. The score is calculated based on cancer type, Plt count, Hb level, erythropoietin drug use, WBC count, and BMI [30]. Score 0 is classified as low-risk, score 1–2 is classified as intermediate-risk, and score ≥ 3 is classified as high-risk. VTE incidence in the derivation and validation cohorts, respectively, was 1.8% and 2% in the intermediate-risk category. The patients in the present study were determined as having intermediate risk. These findings demonstrate that it is difficult to predict VTE onset using the Khorana score alone. Recent research has suggested risk prediction using the Vienna score, which combines D-dimer and P-selectin levels with the Khorana score [31, 32]. Therefore, we included D-dimer and P-selectin levels although they are not typically measured in routine medical examination. A limitation in the research design of the present retrospective study is the measured rates of D-dimer and P-selectin of 52.5% and 0%, respectively. It is necessary to investigate whether the Vienna score is useful in predicting VTE onset following ICI administration, in a prospective study.

In the present study, the rate of CAT onset during ICI administration and the risk factors for CAT onset were analyzed. These data will be useful in preventing CAT-associated diseases from becoming severe during ICI administration. However, because the present study was a retrospective medical record survey, there were several limitations; information considered related to VTE onset such as PS, histology, advanced stage (metastatic), and central venous catheters [33] could not be sufficiently extracted; because the study investigated five cancer types (lung cancer, kidney cancer, stomach cancer, urothelial carcinoma, and malignant melanoma), the effects of pre-treatment could not be determined as pre-treatment differed in each cancer type; and although we attempted to investigate the possibility of a sudden increase in reactivated T cells immediately after ICI administration, this could not be performed because T cell count was measured in extremely few patients. In addition, in the present study, the number of CAT onset cases was small, and a sufficient number of cases could not be identified. In future studies, it will be essential to include a larger number of cases.

## Conclusions

It is crucial to pay attention to possible CAT onset during ICI administration, as is the case with VEGF inhibitors. Before ICI administration, it is important to implement careful monitoring to detect CAT incidence, considering thromboembolic disease history and heart disease history in particular. We recommend establishing frameworks for collaboration with other medical departments to achieve this.

## References

[1] AACR 103rd Annual Meeting 2012-Mar 31-Apr 4, 2012; Chicago, IL

[2] Khorana AA (2010). Venous thromboembolism and prognosis in cancer. Thromb Res.

[3] Blom JW, Doggen CJ, Osanto S (2005). Malignancies, prothrombotic mutations, and the risk of venous thrombosis. JAMA.

[4] Navi BB, Reiner AS, Kamel H (2017). Risk of arterial thromboembolism in patients with cancer. J Am Coll Cardiol.

[5] Khorana AA, Connolly GC (2009). Assessing risk of venous thromboembolism in the patient with cancer. J Clin Oncol.

[6] Seng S, Liu Z, Chiu SK (2012). Risk of venous thromboembolism in patients with cancer treated with Cisplatin: a systematic review and meta-analysis. J Clin Oncol.

[7] Fuchs CS, Tomasek J, Yong CJ (2014). Ramucirumab monotherapy for previously treated advanced gastric or gastro-oesophageal junction adenocarcinoma (REGARD) an international, randomised, multicentre, placebo-controlled, phase 3 trial. Lancet.

[8] Zhu AX, Kang YK, Yen CJ (2019). Ramucirumab after sorafenib in patients with advanced hepatocellular carcinoma and increasedαfetoprotein concentrations (REACH-2):a randomised, double-blind, placebo-controlled, phase 3 trial. Lancet Oncol.

[9] Wilke H, Muro K, Van Cutsem E (2014). Ramucirumab plus paclitaxel versus placeboplus paclitaxel in patients with previously treated advanced gastric or gastro-oesophageal junction adenocarcinoma (RAINBOW): a double-blind, randomised phase 3 trial. Lancet Oncol.

[10] Tabernero J, Yoshino T, Cohn AL (2015). Ramucirumab versus placebo in combination with second-line FOLFIRI in patients with metastatic colorectal carcinoma that progressed during or after firstline therapy with bevacizumab, oxaliplatin, and a fluoropyrimidine (RAISE): a randomised, double-blind, multicentre, phase 3 study. Lancet Oncol.

[11] Garon EB, Ciuleanu TE, Arrieta O (2014). Ramucirumab plus docetaxel versus placebo plus docetaxel for second-line treatment of stage IVnon-small-cell lung cancer after disease progression on platinum-based therapy (REVEL): a multicentre, double-blind, randomised phase 3 trial. Lancet.

[12] Nalluri SR, Chu D, Keresztes R (2008). Risk of venous thromboembolism with the angiogenesis inhibitor bevacizumab in cancer patients: a meta-analysis. JAMA.

[13] Weber J, Mandala M, Del Vecchio M (2017). Adjuvant Nivolumab versus Ipilimumab in resected stage III or IV melanoma. N Engl J Med.

[14] Ferris RL, Blumenschein G, Fayette J (2016). Nivolumab for recurrent squamous-cell carcinoma of the head and neck. N Engl J Med.

[15] Kang YK, Boku N, Satoh T (2017). Nivolumab in patients with advanced gastric orgastro-oesophageal junction cancer refractory to, or intolerant of, at least two previous chemotherapy regimens (ONO-4538-12, ATTRACTION-2): a randomised, double-blind, placebo-controlled, phase 3 trial. Lancet.

[16] Tomita Y, Fukasawa S, Shinohara N (2019). Nivolumab versus everolimus in advanced renal cell carcinoma: Japanese subgroup 3-year follow-up analysis from the phase III CheckMate 025 study. Jpn J Clin Oncol.

[17] Yamazaki N, Kiyohara Y, Uhara H (2017). Efficacy and safety of nivolumab in Japanese patients with previously untreated advanced melanoma: a phase II study. Cancer Sci.

[18] Hida T, Nishio M, Nogami N (2017). Efficacy and safety of nivolumab in Japanese patients with advanced or recurrent squamous non-small cell lung cancer. Cancer Sci.

[19] Nishio M, Hida T, Atagi S (2017). Multicentre phase II study of nivolumab in Japanese patients with advanced or recurrent non-squamous non-small cell lung cancer. ESMO Open.

[20] Maruyama D, Hatake K, Kinoshita T (2017). Multicenter phase II study of nivolumabin Japanese patients with relapsed or refractory classical Hodgkin lymphoma. Cancer Sci.

[21] Varki (2007) A Trousseau's syndrome: multiple definitions and multiple mechanisms. Blood 110:1723–1729 10.1182/blood-2006-10-053736 PMC1976377 17496204

[22] Kunimasa K, Nishino K, Kimura M (2018). Pembrolizumab-induced acute thrombosis: a case report. Medicine (Baltimore).

[23] Johnson DB, Balko JM, Compton ML (2018). Fulminant myocarditis with combination immune checkpoint blockade. N Engl J Med.

[24] Wang DY, Salem JE, Cohen JV (2018). Fatal toxic effects associated with immune checkpoint inhibitors: a systematic review and meta-analysis. JAMA Oncol.

[25] Vasan SK, Rostgaard K, Majeed A (2016). ABO blood group and risk of thromboembolic and arterial disease: a study of 1.5 million blood donors. Circulation.

[26] Pulivarthi S, Gurram MK (2014). Effectiveness of d-dimer as a screening test for venous thromboembolism: an update. N Am J Med Sci.

[27] Pabinger I, Thaler J, Ay C (2013). Biomarkers for prediction of venous thromboembolism in cancer. Blood.

[28] Stein PD, Hull RD, Patel KC (2004). D-dimer for the exclusion of acute venous thrombosis and pulmonary embolism: a systematic review. Ann Intern Med.

[29] Scappaticci FA, Skillings JR, Holden SN (2007). Arterial thromboembolicevents in patients with metastatic carcinoma treated with chemotherapy and bevacizumab. J Natl Cancer Inst.

[30] Khorana AA, Kuderer NM, Culakova E (2008). Development and validation of a predictive model for chemotherapy-associated thrombosis. Blood.

[31] Ay C, Dunkler D, Marosi C (2010). Prediction of venous thromboembolism in cancer patients. Blood.

[32] Thaler J, Ay C, Pabinger I (2012). Venous thromboembolism in cancer patients-risk scores and recent randomised controlled trials. Thromb Haemost.

[33] Zamorano JL, Lancellotti P, Rodriguez Muñoz D (2016). ESC position paper on cancer treatments and cardiovascular toxicity developed under the auspices of the ESC Committee for practice guidelines. (2016) the task force for cancer treatments and cardiovascular toxicity of the European Society of Cardiology (ESC). Eur Heart J.

